# Methylphenidate-Induced Psychotic Symptoms in 65-Year-Old Female with ADHD

**Published:** 2018-10

**Authors:** Elliyeh Ghadrdan, Maryam Mousavi, Padideh Ghaeli

**Affiliations:** 1 Department of Clinical Pharmacy, School of Pharmacy, Tehran University of Medical Sciences, Tehran, Iran.; 2 Research Center for Rational Use of Drugs, Tehran University of Medical Sciences, Tehran, Iran.; 3 Pharmacy Services, Roozbeh Psychiatric Hospital, Tehran University of Medical Sciences, Tehran, Iran.

**Keywords:** *Adult Attention-Deficit Hyperactivity Disorder*, *Methylphenidate*, *Psychosis*

## Abstract

Methylphenidate, a stimulant, is prescribed commonly in the treatment of attention deficit hyperactivity disorder (ADHD) in children and adults. Methylphenidate is generally considered a safe medication, however, some rare adverse effects, such as psychotic symptoms, may occur with its therapeutic or high doses. Additionally, this medication has a potential of abuse, especially among teenagers. There are several published cases regarding methylphenidate-induced psychosis in young adults. However, psychosis due to methylphenidate has been rarely reported in the elderly. This case presents psychotic manifestations due to methylphenidate in a 65-year-old female who was taking this medication for ADHD. She consumed 3 to 4 methylphenidate hydrochloride tablets per day for several months and thought that they were sleeping pills. Antipsychotic medication was initiated and methylphenidate was discontinued which resulted in improvement of her psychosis. Alternative diagnoses, including bipolar mood disorder with psychotic feature or mood disorder due to general medical condition, were ruled out because her psychotic symptoms appeared after taking several methylphenidate tablets and disappeared after discontinuation of this medication.

Methylphenidate is a central nervous system (CNS) stimulant and is commonly prescribed for the treatment of attention deficit hyperactivity disorder (ADHD) in children and adults. Also, it is available in both immediate release and extended release formulations, which are different in half-life and duration of action. 

Methylphenidate is generally considered a safe and tolerable medication although its side effects, including insomnia, headache, anorexia, and gastrointestinal complications, are common. This medication has a potential of abuse, especially among teenagers. The abuse potential of methylphenidate exists due to the euphoric feeling after consuming high doses of this drug (oral, intranasal, or intravenous injection) (1, 2). Using high doses of methylphenidate has been reported to cause serious complications, such as psychosis, seizure, liver damage, and cardiovascular side effects. 

Previous studies have suggested that psychotic symptoms may be found following methylphenidate consumption in patients with ADHD. Most of the related cases have been reported in young patients, especially, shortly after the initiation of methylphenidate consumption. In a case-control study, Auger et al. noted that the occurrence of psychosis in high doses of methylphenidate (≥ 120mg) is significantly higher than its occurrence in standard doses of this medication (14% vs. 3%) (3). 

In another study, Kraemer et al. reported methylphenidate-induced psychosis with usual doses of this medication in 3 patients who did not have any history of psychotic symptoms (4). 

This report presents a 65-year-old female, with a diagnosis of adult ADHD, who was hospitalized due to psychosis after consuming 3 to 4 methylphenidate tablets per day by mistake for 2 months. She was taking methylphenidate for about 15 years prior to this episode without any history of psychosis.

## Case Reports

A 65-year-old female with a history of ADHD and obsessive-compulsive disorder (OCD) was admitted to the psychiatric emergency department with complaint of agitation and unusual behaviors during the past 2 months as stated by her son who accompanied her at the admission. She was an uneducated widowed housewife with 7 children andwas living with one of her sons. The patient’s weight was 60 kg (132 lb) and her height was 166 cm (65.35 inches) at admission. She was cooperative during the mental status examination. Her appearance and make up were appropriate. She was talkative, had high speech rate, and voice volume. No cognitive impairment was detected. According to the patient’s statement, she did not need to sleep for more than 2 to 3 hours every night. Her affect and mood were congruent. During the interview, the patient was experiencing persecutory delusions. She was worried about her food being poisoned and also about being offended by her son. Also, she talked about being raped by her son on different occasions. According to her and her companion’s statements, the patient’s delusional thoughts started about 2 months ago. She had never used alcohol, tobacco, or illicit drugs in the past.

She was diagnosed with OCD and ADHD about 30 years ago. Additionally, she experienced depression after her husband’s death about 15 years ago. She did not have a history of psychotic or manic episodes in the past. She has been treated with fluoxetine 20 mg daily, methylphenidate hydrochloride 10 mg daily, and alprazolam 0.5 mg daily in the last 15 years. Alprazolam had been prescribed because of her sleeping problem and she used it as a sleeping pill. She had no family history of psychiatric disorders. Due to her new clinical manifestations, such as restlessness, irritability, talkativeness, agitation, paranoia, delusions, and sleep disturbance, occurrence of a recent psychotic episode was considered. Bipolar mood disorder with psychotic features was considered as one of the differential diagnosis. However, this diagnosis was ruled out considering that she had no previous history of manic episodes and considering the finding of Valiengo et al. who suggested the prevalence rate of bipolar mood disorder to be very low in older patients (~0.1%–0.5%) (5). 

The patient started taking methylphenidate hydrochloride with a dose of 10 mg per day in the past 15 years without any reported psychotic manifestation on that dose. During the interview, she mentioned that she had taken methylphenidate hydrochloride 3 to 4 times a day by mistake for the last 2 months, thinking she was taking sleeping pills. 

The initial vital signs were as follow: blood pressure 120/80 mm Hg, respiratory rate 15 breaths/minute, pulse rate 60 beats/minute, and oral temperature 37°C. The patient reported that she was diagnosed with hypothyroidism many years ago for which levothyroxine was prescribed. However, she admitted discontinuing levothyroxine on her own without any specific reason in the recent years. She had no documents on her thyroid profile; thus, her thyroid function was evaluated at the center laboratory, and an evidence of hypothyroidism was found. Although the association between hypothyroidism and psychiatric presentation has been documented (6), the patient never showed any psychotic symptoms until this recent episode; therefore, her psychotic symptoms were not thought to be attributed to her thyroid problem. The patient was referred to an endocrinologist to follow-up her thyroid status.

Brain imaging and neurological assessments were also performed but did not show any problems. 

Laboratory findings were as follow: white blood cell count, 5900/mm3; hemoglobin, 13.4 g/dL; platelet count, 215×1000/mm3; 25(OH) D3, 41ng/mL; homocysteine, 14.8 mcmol/L; vitamin B12, 1281pg/mL; folic acid, 8.6 ng/mL; Anti TPO, 600IU/mL; T3 Uptake, 1.1; FT4, 1ng/dL; T4, 8mcgr/dL; T3, 0.9ng/dL; TSH,17.9mcIU/mL. Additionally, urine toxicology was negative for morphine, amphetamine, and methamphetamine. 

Initially, to reduce psychotic symptoms, haloperidol, a high potent antipsychotic, was started at 0.5 mg 2 times a day. After 7 days when her symptoms were improved, this medication was discontinued due to the increased risk of extrapyramidal side effects with haloperidol and olanzapine, with a dose of 5 mg was started. Olanzapine was increased to 15 mg per day over a 2-week period. Furthermore, methylphenidate hydrochloride was discontinued and the patient was observed for any sign of withdrawal. Additionally, levothyroxine 50 mcg was prescribed for the patient. After 7 days of hospitalization, aggression and paranoid ideation decreased and sleeping disturbance improved significantly. Four weeks after starting levothyroxine and olanzapine, her symptoms improved significantly with an exception of mild residual overvaleud idea. At that time, her thyroid profile was T4, 7.7mcgr/dL; T3, 1ng/dL and TSH, 9mcIU/mL. 

## Discussion

Methylphenidate, a stimulant, can be administrated in the treatment of many disorders or symptoms, such as ADHD, narcolepsy, cancer related fatigue, older adults’ major depressive disorder, brain injury, and cognitive disorder (2). Methylphenidate increases the post synaptic dopamine and norepinephrine concentrations by CNS dopamine and norepinephrine reuptake inhibition (7). Besides, it can act as a D1 receptor activator in postsynaptic neuron (8). Insomnia, headache, and decreased appetite are among common side effects observed by this medication (2). 

Psychotic and mania-like manifestation with methylphenidate have been noted in the medical literature. These complications have been reported with both therapeutic dosage or when abused. Most of methylphenidate-induced psychotic symptoms have been reported in children and adolescents. Two case reports by Porfirio et al. and Halevy et al. presented 2 children with ADHD who experienced visual hallucinations after methylphenidate consumption. In both cases, hallucinations resolved after discontinuation of methylphenidate (9, 10). 

Kraemer et al. reported 3 cases with a diagnosis of adult ADHD (29, 38, and 45 years old) who presented with psychotic features while on therapeutic dosage of methylphenidate. One of these cases was a 29-year-old male who was receiving methylphenidate 20 mg/d for several weeks, whose medication dosage was changed to 36 mg of modified-release formulation of methylphenidate. He experienced paranoid psychosis after dosage and formula change. After discontinuation of methylphenidate and initiation of antipsychotics (risperidone, olanzapine, and paliperidone, respectively). \psychotic symptoms were resolved in all the 3 cases during 2 to 21 days (4). 

Hesapcioglu et al. reported 2 cases of children with ADHD (10 and 7.5 years old) with complaint of delusional thoughts. Their symptoms appeared after the first dose of long-acting methylphenidate 36 and 18 mg per day, respectively. The delusional symptoms disappeared completely a few days after discontinuation of methylphenidate. In the first case, methylphenidate was replaced by atomoxetine, but the second patient refused taking another medication (8). 

Ross et al. reviewed psychotic-like symptoms during stimulant therapy in children with ADHD and suggested a risk of psychotic or manic-like symptoms in 0.25% of children treated by stimulants. Out of the 60 cases who were evaluated in that study, in 55 cases psychotic symptoms were resolved after stimulant discontinuation (11). 

On the other hand, methylphenidate has a high potential for being abused, especially in young adults. There is also the possibility of simultaneous use of cannabis and other illicit drugs in young adults on methylphenidate that can increase the risk of psychotic symptoms. Cinosi et al. presented a patient with manic symptoms induced by abusing cannabis and methylphenidate consumed concurrently. Doses of stimulants resulting in psychotic manifestation is variable among abusers. Therefore, adequate evaluation of psychotic symptoms as well as a history of drug abuse were suggested before prescribing stimulants. Furthermore, careful follow- up was also recommended by the authors (12). 

## Conclusion

I this case presented a 65-year-old female with psychotic symptoms due to consumption of up to 40 mg per day of methylphenidate, thinking it was a sleeping pill by mistake. The patient did not show any psychotic symptoms while on her prescription dose of 10 mg methylphenidate per day during the past 15 years. Occurrence of psychosis induced by methylphenidate should be considered in high doses of this medication even though there are reports of psychosis induced by this medication in therapeutic dosages as well. It has been reported that older people are highly vulnerable to the adverse effects of psychotropic medications including psychotic side effects (13). This case emphasizes on giving medication education to the elderly to avoid unwanted adverse effects of medications by providing information on the appropriate use of medications. 
